# Integrated Modeling and Target Classification Based on mmWave SAR and CNN Approach

**DOI:** 10.3390/s24247934

**Published:** 2024-12-12

**Authors:** Chandra Wadde, Gayatri Routhu, Mark Clemente-Arenas, Surya Prakash Gummadi, Rupesh Kumar

**Affiliations:** 1 Department of Electronics and Communication Engineering (ECE), SRM University AP, Amaravati 522502, India; chandra_vadde@srmap.edu.in (C.W.); gayatri_routhu@srmap.edu.in (G.R.); suryaprakash_gummadi@srmap.edu.in (S.P.G.); 2 Wireless Sensing and Imaging Laboratory & 6G Research Laboratory, SRM University AP, Amaravati 522502, India; 3 Electronics Circuits and Systems Research Group, Universidad Nacional Tecnologica de Lima Sur UNTELS, Villa El Salvador, Lima 15834, Peru; mclemente@untels.edu.pe

**Keywords:** mmWave FMCW radar, synthetic aperture radar (SAR), reflectivity-added images, convolutional neural networks (CNN)

## Abstract

This study presents a numerical modeling approach that utilizes millimeter-wave (mm-Wave) Frequency-Modulated Continuous-Wave (FMCW) radar to reconstruct and classify five weapon types: grenades, knives, guns, iron rods, and wrenches. A dataset of 1000 images of these weapons was collected from various online sources and subsequently used to generate 3605 samples in the MATLAB (R2022b) environment for creating reflectivity-added images. Background reflectivity was considered to range from 0 to 0.3 (with 0 being a perfect absorber), while object reflectivity was set between 0.8 and 1 (with 1 representing a perfect electric conductor). These images were employed to reconstruct high-resolution weapon profiles using a monostatic two-dimensional (2D) Synthetic Aperture Radar (SAR) imaging technique. Subsequently, the reconstructed images were classified using a Convolutional Neural Network (CNN) algorithm in a Python (3.10.14) environment. The CNN architecture consists of 10 layers, including multiple convolutional, pooling, and fully connected layers, designed to effectively extract features and perform classification. The CNN model achieved high accuracy, with precision and recall values exceeding 98% across most categories, demonstrating the robustness and reliability of the model. This approach shows considerable promise for enhancing security screening technologies across a range of applications.

## 1. Introduction

The advancement of radar technology, particularly in the millimeter-wave (mm-Wave) frequency bands spanning from 30 GHz to 300 GHz, has significantly expanded the capabilities of high-resolution imaging across diverse applications, including automotive safety [1,2], healthcare monitoring [3,4,5], and, most notably, security and surveillance [6,7,8]. The unique penetration capabilities of mm-Wave radar, which allow it to pass through materials like clothing, ceramics, and luggage without compromising privacy [9,10,11], make it a powerful tool for non-invasive inspection. Among the various types of radar systems, mm-Wave Frequency-Modulated Continuous Wave (FMCW) radars, particularly those operating in the 77 GHz to 81 GHz range, have demonstrated exceptional efficacy in generating high-resolution images crucial for precise object recognition and classification [12,13,14]. This ability to deliver detailed imagery, even in challenging environments [15,16,17], positions mm-Wave FMCW radar as a critical technology for advanced imaging applications, enhancing both the resolution and reliability of security screening and surveillance systems [18,19,20].

Previous works in the field of image reconstruction using mm-Wave FMCW radar and SAR techniques have primarily focused on enhancing the accuracy and robustness of target detection and classification by leveraging advanced radar signal processing and deep learning models. For instance, refs. [6,7] utilized a broad frequency range from 76 GHz to 81 GHz and integrated Deep Residual Networks (ResNet) to classify targets based on Synthetic Aperture Radar images. Their approach benefited from ResNet’s deep architecture, which is particularly adept at extracting complex features from high-dimensional radar data, thereby improving classification accuracy, especially in challenging conditions involving noise and clutter. However, the complexity of the network also meant increased computational demands, data size and processing times, which could limit real-time applicability. Similarly, ref. [21] explored the use of SAR imaging combined with deep learning techniques for high-resolution radar image reconstruction. This work emphasized enhancing the resolution and clarity of SAR images to better identify objects, employing complex neural networks to manage the intricacies of radar signal patterns, while effective in improving image quality, the approach required extensive data preprocessing and high computational power, which can be a drawback for real-time or resource-constrained applications.

Other studies, such as those by [11,22], also focused on improving feature extraction and classification accuracy through advanced neural network architectures like ResNet. These networks were trained on large datasets to generalize well across various scenarios, including different object types and environmental conditions. However, such methods often involved broader frequency bands and more layers in the network, contributing to greater computational complexity and making them less feasible for real-time deployment in security screening applications.

Data creation for machine learning models, particularly in the context of mm-Wave FMCW radar and SAR imaging, poses significant challenges, as highlighted in existing research. From previous studies, we have underscored the difficulties in generating and curating datasets that are both comprehensive and representative. These complexities stem from the need to collect high-quality radar images under various real-world conditions, including different environments, angles, and object orientations, which often necessitate extensive manual efforts in collection and labeling. Additionally, simulating realistic radar reflectivity for diverse materials and surfaces further complicates the data creation process, requiring advanced preprocessing and augmentation techniques to produce reliable training datasets. The scarcity of publicly available radar image datasets exacerbates these challenges, forcing reliance on synthetic data or limited real-world samples that may not fully capture the intricacies of actual environments.

This model addresses these challenges by introducing an innovative data creation approach that integrates 1000 images of five different weapons—grenades, knives, guns, iron rods, and wrenches—sourced from online resources and processed with realistic reflectivity simulation. By narrowing the frequency range to optimize radar performance and employing SAR imaging techniques, our study offers a more streamlined and efficient solution for generating high-quality training data. Using MATLAB, 3605 reconstructed images are generated and stored for further processing. The CNN algorithm is chosen for its exceptional ability to automatically learn and extract complex features from high-resolution images, making it ideal for accurately classifying these reconstructed mm-Wave FMCW radar images.

The primary contributions of this research work are summarized as follows:

1. Reflectivity Mapping and Standardization of Online Data Set: This study involves collecting 1000 online images of weapons from various resources and generating 3605 samples, with reflectivity mapping and standardization applied during preprocessing phase. These reflectivity-added images are used for further image reconstruction using SAR imaging techniques.
2. Utilization of mm-Wave FMCW Radar with 2D SAR Imaging: Preprocessed images are reconstructed using mm-Wave FMCW radar combined with 2D SAR imaging techniques.
3. ML Classification Approach: The reconstructed images are classified using CNNs, enhancing classification accuracy.
4. Complete Analytical Approach: The approach employs SAR imaging for high-resolution reconstruction of preprocessed weapon images and CNNs for accurate classification. It achieves over 98% accuracy, offering a non-invasive, high-resolution alternative to traditional security screening methods with enhanced capabilities to detect weapons through non-metallic surfaces.

The hardware configuration included an NVIDIA GeForce RTX 3060 for accelerating the training process of the Convolutional Neural Network. A high-performance CPU with 32 GB RAM and 1.5 TB storage running Windows 11 Pro was used to handle demanding data preprocessing tasks. Despite these powerful tools, integrating the image reconstruction and classification workflows seamlessly proved to be a complex and demanding process.

The remainder of this paper is organized as follows: Section 2 provides an overview of the framework, detailing the foundational elements and methodologies. Section 3 describes data collection and preprocessing techniques, focusing on generating reflectivity-added images. Section 4 presents the modeling of mm-Wave FMCW radar, including specific parameters and configurations. Section 5 describes the machine learning technique used for image classification, specifically the CNN algorithm. Section 6 discusses the results and offers a comprehensive analysis. Section 7 compares the results with previous works, highlighting the advancements and contributions of this study. Finally, Section 8 concludes the paper, summarizing the key outcomes and suggesting directions for future research.

## 2. Framework Overview

**Figure 1:**
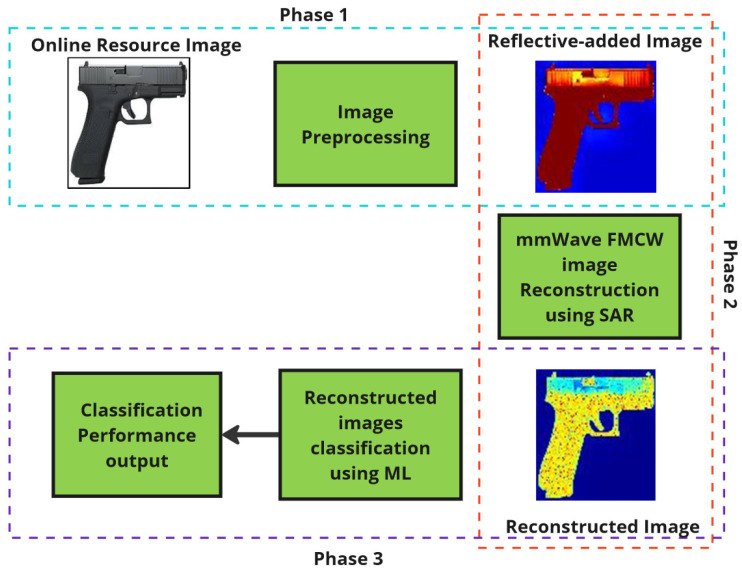
Modeling mm-Wave Imaging.

The research work is outlined in three phases, as shown in Figure 1. Phase 1 covers online image gathering and preprocessing, Phase 2 explains the SAR imaging technique for reconstructing images, and Phase 3 provides details on the CNN classification of reconstructed images. This process is meticulously designed to enhance the accuracy and reliability of security screening technologies, addressing the limitations of traditional methods. The workflow is summarized in Table 1.

### 2.1. Phase 1: Image Preprocessing

The first phase begins with acquiring images of different weapons from various online resources, representing the initial input for the system. This study collected 1000 diverse images with varying dimensions, such as size and color. The primary goal of this phase is data collection, reflectivity mapping, and standardization of online resource images in uniform size. Preprocessing is crucial for preparing raw data for subsequent analysis. The collected images undergo preprocessing steps including resizing, normalization, and possibly contrast enhancement to ensure they are optimized for further processing. This step is vital as it enhances image quality, ensuring that the subsequent processes can be carried out effectively. The output of this phase is a reflectivity-added image, which incorporates reflectivity data to simulate how the object would interact with radar signals.

### 2.2. Phase 2: mm-Wave FMCW Image Reconstruction Using SAR

The second phase focuses on using the preprocessed images for image reconstruction via mm-Wave FMCW radar combined with SAR imaging techniques. The reflectivity-added images generated in Phase 1 are processed in this stage. Here, the SAR imaging technique is applied to reconstruct a high-resolution image that mimics the radar’s interaction with the object. SAR is a form of radar used to create detailed images or maps of objects or landscapes. It operates by sending out radar pulses and capturing the echoes that return after bouncing off the object. The phase shifts in the returning signals are analyzed to produce a high-resolution image. The reconstructed image produced by this phase is critical as it represents a synthetic view of the object, mimicking how the mm-Wave FMCW radar would capture it—crucial for accurate object detection and classification in the next phase.

### 2.3. Phase 3: Classification Using Machine Learning

The third phase focuses on classifying the reconstructed images using machine learning algorithms in a Python (3.10.14) environment. The reconstructed image obtained from Phase 2 serves as the input to a machine-learning model. CNN algorithm is chosen for this study due to good performance in image feature extraction and classification. Because CNNs can learn and extract features from input images using multiple layers of convolution and pooling, they are very effective in image classification tasks. In this phase, the CNN analyzes the reconstructed image, identifying patterns and features that correspond to specific classes of objects. For instance, in security applications, the CNN would be trained to distinguish between different types of weapons or other objects of interest. The final output of this phase is the classification performance, which indicates the effectiveness of the CNN in accurately identifying and categorizing the objects based on the reconstructed radar images.

## 3. Data Collection and Preprocessing to Generate Reflectivity-Added Image

This work gathered online images of different weapons from various resources. The choice to use online resource images as the base for generating reflectivity-added images was driven by the need for a comprehensive and varied dataset that reflects real-world conditions. These gathered online resource images are preprocessed and utilized for reconstruction in further process.

**Figure 2:**
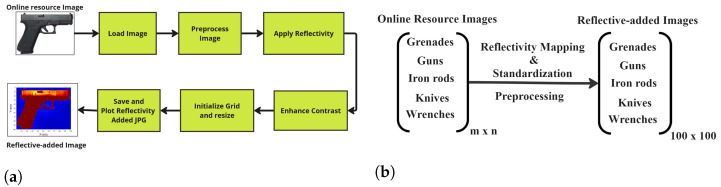
(**a**) Proposed workflow for reflectivity mapping and size standardization and (**b**) Reflectivity Mapping and size standardization: Preprocessing.

Figure 2a illustrates a detailed workflow for generating reflectivity-added images from online resource images. This represents an important step in preparing data for mm-Wave FMCW radar imaging and classification tasks. The process begins with loading of the online resource image, which serves as the raw input for the entire procedure. This image, consisting of an object such as a weapon, is then subjected to a series of preprocessing steps. Preprocessing is essential for ensuring that the data is in a suitable format for the application of reflectivity. These images are preprocessed to generate reflectivity-added images followed by resizing, and normalizing pixel values are employed to create a uniform and clean image, reducing any potential artifacts that could interfere with later stages of the process.

In the preprocessing step, reflectivity is added, which simulates how radar signals would interact with the object depicted in the image. Reflectivity is a measure of the mm-Wave FMCW radar signal reflected back from the object, and this step converts a regular photographic image into a format usable for mm-Wave FMCW radar-based analysis. Specifically, the reflectivity ρ is expressed mathematically as:

(1) $$\rho \left(x,y\right)=\frac{I(x,y)}{I_{\max}}$$

where I(x,y) represents the pixel intensity at position (x,y), and $I_{\max}$ is the maximum pixel intensity in the image. This normalization step ensures that reflectivity values fall within a range suitable for simulation. Following this, contrast enhancement is applied to improve the visibility of features in the image, ensuring that differences in reflectivity across the object are more pronounced. This step is particularly important for objects with subtle reflectivity variations, making it easier to identify and analyze key features.

The workflow then proceeds to initialize a grid and resize the image. Initializing a grid involves overlaying a grid structure onto the image, which helps in accurately mapping the spatial properties of the object within the image. This step, along with resizing the image of different dimensions of ‘m × n’ to standard dimensions of grid size ‘100 × 100’ is shown in Figure 2b. To scale the object image to match the grid size, the following interpolation formula is generally used:

(2) $$RI=interp(OriginalImage_{m\times n},NewSize_{100\times 100})$$

where RI represents Resized Image, NewSize is modeled grid_size.

The resulting images, now embedded with reflectivity data, are saved in a labeled folder as JPEG format and plotted to provide a visual representation of how the object will appear under reflectivity property conditions. These plotted images serve as the input for the subsequent phases of mm-Wave FMCW radar image reconstruction and classification using the CNNs algorithm.

**Figure 3:**
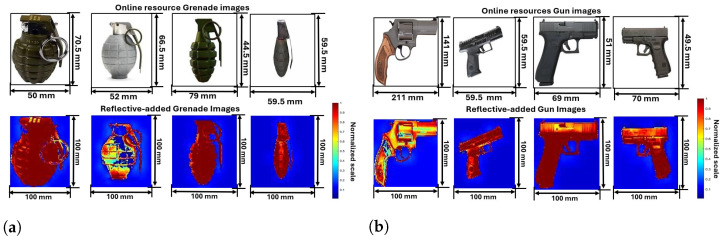
Online resource and preprocessed reflectivity-added images based on proposed method (**a**) Grenades; (**b**) Guns.

**Figure 4:**
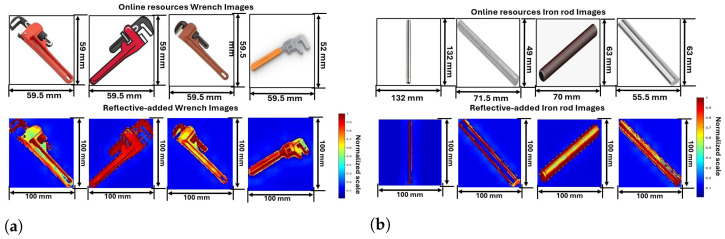
Online resource and preprocessed reflectivity-added images based on proposed method (**a**) Wrenches; (**b**) Iron-roads.

**Figure 5:**
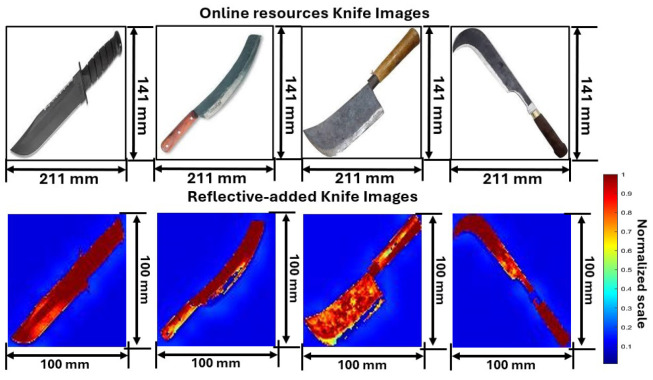
Online resource and preprocessed reflectivity-added knife images based on proposed method.

Five distinct weapon images from online resources images and reflectivity-added images after preprocessing are shown in Figure 3, Figure 4 and Figure 5. Out of a total of 1000 images, some random images are chosen to analyze and understand the process. The parameters of reflectivity-added images considered in this study are shown in Table 2.

The first set of Figure 3a images represents grenades. The top row presents different types of grenades sourced from online resources, each labeled with their respective dimensions. The first grenade measures 70.5 mm in height and 50 mm in width, featuring a typical ribbed surface. The second grenade, with dimensions of 66.5 mm by 52 mm, has a smooth cylindrical body with a prominent safety pin and lever. The third grenade, measuring 44.5 mm by 79 mm, is compact and oval-shaped, often used for smoke or flash purposes. The fourth grenade, at 59.5 mm by 59.5 mm, has a streamlined design for specific tactical applications.

The comparison of the online resource images of grenades with the corresponding reflectivity-added images after preprocessing is shown in Figure 3a. This preprocessing step ensures that the object has high reflectivity while the background has low reflectivity. The added reflectivity highlights the metallic and structural features of the grenade. The first reflectivity image shows high reflectivity on the ribbed surface, with the grenade appearing mostly red, indicating strong reflections. The second reflectivity image reveals a more complex pattern due to the cylindrical shape and metallic surface of the grenade, with variations in color reflecting different angles and surfaces interacting with the radar signals. The third reflectivity image, representing the compact oval-shaped grenade, shows a relatively uniform reflectivity, with distinct reflections from the safety pin and lever. The fourth reflectivity image of the streamlined grenade exhibits high reflectivity on its body, with some areas reflecting less due to their orientation or material properties.

Likewise, the second weapon, the gun, focuses on online resource images and reflectivity-added images comparison. The analysis of gun images, showcasing their original appearances and corresponding preprocessed reflectivity mapping properties. The top row displays images of various guns sourced from online resources, with dimensions labeled. The first gun is a revolver measuring 211 mm in length and 141 mm in height, featuring a wooden grip and metallic barrel. The second is a compact semi-automatic pistol, 59.5 mm in length, known for its lightweight and ease of concealment. The third gun is a larger semi-automatic pistol, 69 mm in length and 51 mm in height, commonly used by military and police forces. The fourth is a subcompact pistol, 70 mm in length and 49.5 mm in height, ideal for concealed carry.

The second row in Figure 3b shows the reflectivity-added images of guns. Similar to the grenade images, the reflectivity values were added during preprocessing to emphasize the gun’s structural components. The high reflectivity of the object allows for the detailed contours of the gun to be captured effectively, while the background remains less reflective. The revolver’s metallic barrel exhibits high reflectivity, shown in red, and wooden grip in yellow tale, indicating strong reflections. The compact pistol shows a complex reflectivity pattern due to its polymer frame and metallic components. The larger semi-automatic pistol demonstrates relatively uniform reflectivity, with high reflectivity areas highlighting its metallic parts. The subcompact pistol displays high reflectivity on its body, with some variations due to orientation or material properties.

The third set of images illustrates wrenches. Figure 4a presents a detailed analysis of wrench images, showing their original appearances and corresponding reflectivity properties. The first row represents images of various wrenches sourced from online resources, each labeled with their respective dimensions. The first wrench, measuring 59.5 mm in length, is a standard adjustable wrench with a red handle. The second wrench, also 59.5 mm, features an adjustable jaw with a black handle and red body. The third wrench, characterized by a wooden handle and metallic jaw, offers a different ergonomic grip. The fourth wrench, fully metallic and 59.5 mm in length, is designed for heavy-duty applications. The fifth wrench, slightly smaller at 52 mm, has a metal body with a plastic-coated handle for lighter tasks.

The reflectivity-added wrench images give detailed reflective values that are added to highlight the object’s features, such as the metallic parts of the wrench. The uniformity in the object’s reflectivity ensures that the wrench’s shape and structure are well-defined. The background, with lower reflectivity, fades into insignificance, making the wrench stand out in the processed images. Similar to grenades, reflectivity-added properties were observed in previous cases. The first wrench displays high reflectivity along its handles and jaws, indicated by red. Yellow tale color at the middle where the material has different shade effects. The second wrench has high reflectivity compared to the first wrench showing that it is the perfect material. The third wrench’s wooden handle and metallic jaw create a unique reflectivity pattern, reflecting different materials’ interactions. The fourth wrench shows high reflectivity throughout its fully metallic body, highlighting its uniform surface. The fifth wrench displays distinct reflectivity variations due to its plastic-coated handle, with metal parts showing higher reflectivity.

The fourth set focuses on iron rods. Figure 4b presents a detailed analysis of iron rod images, comparing both their original and corresponding reflectivity properties. The images of various iron rods are sourced from online resources, each labeled with their respective dimensions. The first rod, 132 mm in length, is a plain cylindrical metal rod. The second rod, measuring 71.5 mm, features a smooth metallic surface. The third rod, at 70 mm, has a threaded surface for fastening purposes. The fourth rod, 63 mm in length, is another smooth cylindrical rod, while the fifth rod, 55.5 mm in length, has a textured surface for increased grip.

The second row in the fourth set of images shows reflectivity-added iron rods, where reflective values were added to enhance the object’s visibility in the SAR imaging process. The preprocessing reflects the high reflectivity of the metal rod while keeping the background reflectivity low. This distinction between the object and background ensures that the iron rod’s structural integrity and details are preserved and highlighted in the SAR-reconstructed images. The first and second rods display high, uniform reflectivity along their lengths, indicating consistent surface interactions, in the middle of these two images are considered as background reflectivity due to shade replication. The third rod, with its threaded surface, shows a complex reflectivity pattern with alternating high and low bands due to the thread ridges. The fourth rod exhibits a similar uniform reflectivity to the third rod, while the surface results in distinct reflectivity variations.

The final set of images represents knives. Knife images are thoroughly analyzed in Figure 5, which also displays the image’s original appearances and preprocessed reflectivity characteristics. The first knife, measuring 211 mm in length and 141 mm in height, is a large combat knife with a serrated edge. The second knife, with the same dimensions, features a curved blade and a wooden handle, often used for agricultural tasks. The third knife is a cleaver with a wide, heavy blade, commonly used in culinary applications. The fourth knife is a machete with a long, curved blade for cutting through vegetation. The fifth knife, also 211 mm in length, has a hooked blade used for slicing motions in tasks like pruning.

The reflectivity-added knife images with high reflectivity assigned to the knife ensure that its sharp edges and metallic surfaces. The low-reflectivity background helps isolate the knife, making it the focal point of the image, which is essential for accurate object recognition and classification in subsequent analysis. The first knife’s blade and handle show high reflectivity, indicated by red and yellow colors, suggesting consistent surface interaction. The second knife displays high reflectivity along its curved blade and handle, reflecting its distinctive shape. The third knife’s cleaver blade creates a unique pattern due to its wide, heavy blade. The fourth knife’s machete blade shows a consistent high reflectivity pattern, highlighting its uniform surface. The fifth knife’s hooked blade displays distinct reflectivity variations, with high reflectivity in metal parts and less reflection in curved areas.

The reflectivity-added images across all object categories provide a detailed understanding of the reflectivity of different materials when mm-Wave FMCW radar interacts with various surfaces and materials. High reflectivity areas indicated by red (0.8 to 1 as perfect material) show strong mm-Wave FMCW radar signal reflections, while lower reflectivity areas indicated by blue color (0 as perfect absorber) indicate weaker reflections. These variations are influenced by factors such as surface texture, material properties, and the angle of incidence. A total 3605 of reflective mapped and size standardization images are generated and saved for SAR reconstruction in the next phase.

## 4. mm-Wave FMCW Radar Modeling

**Figure 6:**
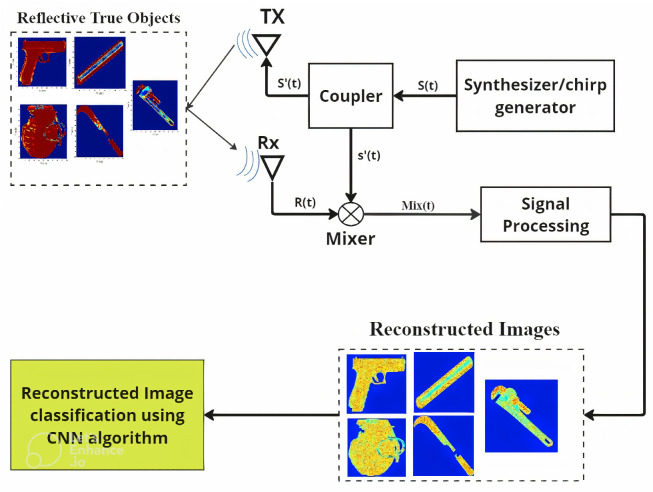
Overall work Process.

In order to reconstruct high-resolution images of different weapons, this research modeled a comprehensive modeling process using mm-Wave FMCW radar. The process included adding reflectivity properties to images and simulating SAR imaging. The provided block diagram Figure 6 illustrates the process of modeling mm-Wave FMCW radar for image reconstruction and classifying weapons using a CNN algorithm. In order to understand the diagram, which shows different transmitters (Tx) and receivers (Rx) without taking into account any space between them, a monostatic SAR configuration is used. The reflectivity-added images are the targets for the mm-Wave FMCW radar to reconstruct, as shown in Figure 6.

The process starts with the Synthesizer/Chirp Generator, which generates the frequency-modulated continuous wave (FMCW) signal [1,23,24,25,26,27,28], S(t). A coupler/splitter then splits the generated signal into two equal parts: the transmitted signal S(t) and the reference signal $S^{\prime }\left(t\right)$.

The Transmitter (Tx), equipped with a high-gain directional antenna, sends the FMCW signal S(t) towards the target area. The signal reflects off the objects and is captured by the Receiver (Rx) as a backscattered signal [23,24], which also uses a high-gain directional antenna to minimize noise and accurately capture the reflected signals. The backscattered signal carries important information about the target’s range and shape in terms of reflectivity. The reflected signal R(t) is mixed with the reference signal $S^{\prime }\left(t\right)$ in the Mixer, producing an intermediate frequency (IF) signal [23,24,26] Mix(t).

To process the IF signal, an analog-to-digital converter (ADC) digitizes it, converting the analog signals into a digital format suitable for further processing by the digital signal processor (DSP) [25,27,28]. The DSP undertakes real-time signal processing tasks, which include filtering, performing Fast Fourier Transform (FFT) operations [26,28,29,30], and extracting range and shape information from the beat frequencies.

The Reconstructed Images section displays the images reconstructed using the mm-Wave FMCW radar data, showing the detected shapes and details of the objects. These reconstructed images are then classified using the CNN Algorithm. The CNN algorithm processes these images to classify the objects, leveraging the spatial hierarchies of the features learned from the mm-Wave FMCW radar images. This integration of mm-Wave FMCW radar with advanced machine learning techniques demonstrates an effective method for weapon detection and classification.

### 4.1. SAR Scanning Area

**Figure 7:**
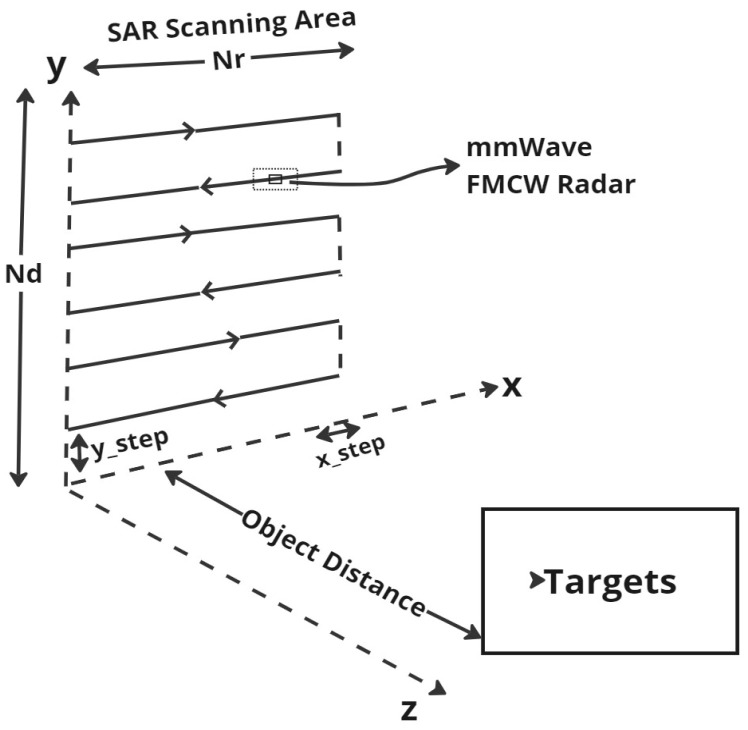
Modeled 2D SAR scanning area.

This work utilized a monostatic SAR configuration [23,24], where the transmitter and receiver are co-located, simplifying hardware design and data processing algorithms. The 2D SAR scanning area is reflected in Figure 7.

The 2D SAR imaging scanning area refers to the specific space within which the radar system operates to reconstruct object shapes. This area serves as the target scene for the radar, determining the range and coverage of the radar signals. In this study, MATLAB is used to model a 2D SAR scanning scenario as shown in Figure 7. The grid size in the xy-plane is defined by Nd and Nr, with the transmit signal directed towards the target within the scanning area, and the step sizes in the xy-plane designated as the x-step and y-step. This approach allows us to comprehensively explore the SAR imaging process and its parameters.

### 4.2. Numerical Modeling Approach

The proposed numerical modeling and analysis method integrates mm-Wave radar imaging using the SAR imaging technique with CNN to classify the reconstructed images. The main focus is on detailing the mathematical formulations, analysis procedures, and computational techniques employed to validate the effectiveness of the model.

The mm-Wave FMCW radar parameters such as carrier frequency, chirp bandwidth, chirp time, and chirp slope are defined based on the radar system specifications. These parameters influence the mm-Wave radar signal’s characteristics and its interaction with objects in the scanning area. A grid is established to simulate the mm-Wave radar’s field of view, with scanning points and step size in both xy-planes corresponding to different spatial locations within the grid as shown in Table 3.

For each grid point, the reflectivity was calculated based on the property of the object from the mm-Wave FMCW radar and the object’s location, providing a detailed reflectivity profile of the scene. The transmitted and received mm-Wave radar signals are modeled for each grid point, incorporating the effects of object reflectivity and range. These signals are processed using a two-dimensional Fast Fourier Transform (2D FFT) to reconstruct the image, effectively representing the object’s reflectivity profile.

To reconstruct the spatial image of the target, the 2D FFT transforms the frequency-domain representation of the received radar signal into the spatial domain. Specifically, the range information is extracted along one dimension (frequency axis), while the Doppler shifts, which provide velocity information, are extracted along the orthogonal dimension. The inversion of this data into spatial coordinates leverages the relationship between the time delay $d_{T}$ and the range of the object, expressed as [6,8]:

(3) $$d_{T}=\frac{2R}{c}$$

where *R* is the range_of the object and *c* is the speed of light. By performing the 2D FFT on the mixed signal, the spatial distribution of the object’s reflectivity is obtained, effectively reconstructing the image of the target.

### 4.3. Tx and Rx Signal Modeling

The transmitted signal (Tx) and the received signal (Rx) are calculated based on the chirp parameters and the range values. The mathematical expression for the transmitted signal is derived as:

(4) $$\begin{array}{c} Tx\left(x,y,z\right)=\sum_{x=1,y=1}^{x=100,y=100}\sum_{f_{i}=1}^{f_{i}=10}\exp\left(j\left(2\pi \left(f_{i}\cdot t_{y}+\frac{S\cdot {\left(t_{y}\right)}^{2}}{2}\right)\cdot \frac{R{\left(x,y,z\right)}^{2}}{c}\right)\right) \end{array}$$

where $f_{i}$ represents the carrier frequency of the radar signal, which is incremented in steps of 0.1 GHz within a 1 GHz bandwidth, ranging from 77 GHz to 78 GHz. $t_{y}$ represents the time variable corresponding to the $y^{th}$ position. The chirp slope, which determines the rate of frequency change over time, is denoted by *S*. The chirp slope represents the frequency modulation over time squared is determined by $S\cdot {\left(t_{y}\right)}^{2}$. R(x,y,z) represents the range to the target at coordinates (x,y,z). It determines how far the target is from the radar system. *z* represents the fixed range of the object from radar. The backscattered signal is derived as:

(5) $$\begin{array}{cc} Rx(x,y,z) & =\sum_{x=1,y=1}^{x=100,y=100}\sum_{f_{i}=1}^{f_{i}=10}\alpha \left(\exp\left(j\cdot 2\pi \left(f_{i}\cdot \left(t_{y}-T_{d}\right)+\frac{S\cdot {\left(t_{y}\right)}^{2}-T_{d}}{2}\right)\cdot \frac{R{\left(x,y,z\right)}^{2}}{c}\right)\right) \end{array}$$

where, the received signal Rx(x,y,z) represents the complex exponential form of the signal at position (x,y,z). The time delay is represented by $T_{d}$. The chirp slope, which determines the rate of frequency change over time, is denoted by *S*. The chirp slope represents the frequency modulation over time squared is determined by $S\cdot {\left(t_{y}\right)}^{2}$. The entire expression within the exponential function represents the phase of the received signal modulated by the range to the target.

The Mixer combines transmitted and received signals, that generate an intermediate frequency (IF) or beat frequency which is defined as follows.

(6) $$Mix\left(x,y,z\right)=\sum_{x=1,y=1}^{x=m,y=m}\sum_{f_{i}=1}^{f_{i}=10}\alpha \left(x,y,z\right)\cdot Tx\left(x,y,z\right)\cdot Rx\left(x,y,z\right)$$

where Mix(x,y) refers to the complex-valued signal at the grid position (x,y). It represents the accumulated mixed signal at that specific point in the grid. α(x,y) represents the presence of an object in the scanning area at position (x,y). The mixed signal is then processed using a 2D FFT to transform it from the time domain to the frequency domain, followed by an inverse FFT to reconstruct the image in the spatial domain. Finally, the matched filter is designed to filter the backscattered signal. The matched filter is expressed as:

(7) $$MF=\exp(j2\pi f_{c}t)$$

where MF is the matched filter.

## 5. Machine Learning Technique to Classify Reconstructed Images

ML algorithms are adept at analyzing and interpreting complex data patterns, facilitating data-driven decisions and predictions [31,32]. Among these algorithms, deep learning, particularly using neural networks with multiple layers, has shown significant potential [33]. Convolutional Neural Networks (CNNs), a subset of deep learning models, are specifically designed for image classification tasks [34]. Consequently, CNNs are employed in this work to classify images reconstructed from mm-Wave SAR data.

### Convolutional Neural Network Architecture

**Figure 8:**
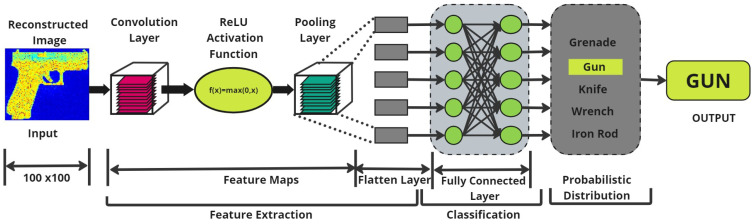
CNN Network Architecture for mm-Wave SAR Image classification.

The CNN architecture employed in this research work, illustrated in Figure 8, is tailored for the classification of reconstructed mm-Wave SAR images and for extracting pertinent features. The network comprises multiple layers, including convolutional layers, activation functions, pooling layers, and fully connected layers [35]. Each layer serves a distinct function, progressively refining the data extracted from raw input images, thereby enabling the recognition and categorization of various object shapes [36]. The figure also outlines the data flow within the network, illustrating how input data is systematically transformed into patterns that the model can interpret. This comprehensive representation provides insight into CNNs’ ability to decipher complex radar images, demonstrating their effectiveness in analyzing and interpreting intricate visual data [37].

The input to the CNN consists of images with dimensions of 100 × 100, which are processed to extract features from grayscale images resized to 64 × 64 [22]. The architecture starts with a convolutional layer that has 32 filters of 3 × 3, utilizing the Rectified Linear Unit (ReLU) activation function [38], which is mathematically defined as:

(8) f(x)=max(0,x)

Here, f(x) represents the output of the ReLU function, where *x* is the input resulting from a convolution operation.

This initial convolutional layer is critical for capturing fundamental features such as edges and textures from the input images [39]. Following the convolutional layer, a max pooling layer with 2 × 2 pool size of is used to down-sample the feature maps, effectively diminishing their spatial dimensions while retaining crucial information [40]. The max pooling operation is expressed as:

(9) P(i,j)=max{I(x,y)∣x,y∈poolregion}

where:

- P(i,j) is the output at (i,j) in the pooled feature map.
- I(x,y) is the input value at position (x,y) in the input feature map (before pooling).
- (x,y)∈poolregion denotes that the coordinates (x,y) are within the specified pooling region, typically a small square region, such as 2 × 2 or 3 × 3.

Max pooling functions by selecting the maximum value within a designated region (pool region) from the input feature map and outputting it. This operation is repeated across the entire input feature map, reducing its size while preserving the most salient features.

The network progresses to a second convolutional layer with 64 filters of size 3 × 3, also employing the ReLU activation function. This layer builds upon the initial features, abstracting and combining them into more complex patterns [41]. Another max pooling layer follows, further down-sampling the feature maps. The architecture also includes a third layer of convolution with 64 filters of 3 × 3 size, designed to extract high-level features and intricate patterns from the data.

The convolutional and pooling layers are followed by the flattening of the feature maps into a one-dimensional vector. A dense, fully connected layer with 64 neurons and ReLU activation [42] then processes this vector. The network can discover intricate connections between the extracted features thanks to this step. The dense layer operation can be described as:

(10) y=Activation(W·x+b)

where:

- *y* is the output vector after applying the activation function.
- *W* is the weight matrix, containing the learned weights during training.
- *x* is the input vector, typically a flattened feature vector from the preceding layer.
- *b* is the bias vector, which is added to the weighted sum to enhance model fitting by shifting the activation function.
- Activation is the function applied to each element of the weighted sum plus bias, introducing non-linearity into the model (common functions include ReLU, sigmoid, and softmax).

The fully connected layer multiplies the input vector *x* by the weight matrix *W*, adds a bias vector *b*, and then applies an activation function to yield the output *y* [42,43] The network can learn through this process, intricate patterns by integrating the learned features non-linearly, thereby capturing more complex relationships within the data.

The final dense layer, consisting of a number of neurons equal to the dataset’s class count and employing the softmax activation function, generates class probabilities [37]. The softmax function is defined as:

(11) $$\sigma {\left(z\right)}_{i}=\frac{e^{z_{i}}}{\sum_{j}e^{z_{j}}}$$

where:

- $\sigma {\left(z\right)}_{i}$ denotes the softmax output for the *i*-th class, representing the probability that the input belongs to class *i*.
- $e^{z_{i}}$ is the exponential of the *i*-th element of the input vector *z*.
- $\sum_{j}e^{z_{j}}$ is the normalization factor that makes sure all of the output probabilities add up to one. It is the sum of exponentials for each element in the input vector *z*.

The softmax function transforms the raw, unnormalized scores in the input vector *z* into a vector of probabilities that sum to 1. This transformation is particularly advantageous for classification tasks, where the output represents the likelihood of each class.

The overall architecture enables the model to progressively learn hierarchical features, advancing from simple to complex, thereby achieving effective classification performance [43].

CNN architecture is specifically designed to analyze pixel data and identify patterns in images [44]. The core component of a CNN is its convolutional layers, which, upon applying several filters to the input image, generate feature maps that capture details such as shapes, textures, and edges through convolution operations [45]. This operation is mathematically represented as:

(12) $$\left(I*K\right)\left(i,j\right)=\sum_{m}\sum_{n}I\left(i-m,j-n\right)\cdot K\left(m,n\right)$$

where:

- The output of the convolution operation is represented by (I∗K)(i,j) at position (i,j) in the output feature map.
- The input image or feature map value at position (i−m,j−n) is indicated by I(i−m,j−n).
- The convolution kernel (filter) value at position (m,n) is represented by K(m,n).
- The summation across all positions of the convolution kernel is represented by $\sum_{m}\sum_{n}$.

This equation encapsulates the fundamental convolution operation within a CNN. It involves sliding the kernel *K* over the input *I* and calculating the dot product at each position, summing the results to form the output feature map’s value at the corresponding position. This process is critical for extracting features necessary for image recognition and classification tasks.

Following convolution, the network introduces non-linearity through activation functions like ReLU, which zero out negative values in the feature map [46]. Pooling layers, often employing max pooling, then reduce the feature maps’ spatial dimensions, minimizing computational costs and preventing overfitting [47].

The feature maps are eventually flattened into a one-dimensional vector and fed into fully connected layers, where each neuron connects to every neuron in the preceding layer, allowing for complex feature extraction and downsampling [37]. The final dense layers typically output a probability distribution across all potential classes using the softmax activation function [48,49].

CNNs are trained using labeled datasets, utilizing backpropagation and gradient descent to optimize [50] their parameters. During the training process, a loss function—typically categorical cross-entropy—that gauges the difference between the actual label distribution and the anticipated probability distribution must be minimized. The definition of the cross-entropy loss is:

(13) $$L\left(y,\hat{y}\right)=-\sum_{k}y_{k}\log\left({\hat{y}}_{k}\right)$$

where:

- The cross-entropy loss between the true labels *y* and the predicted probabilities $\hat{y}$ is represented by $L(y,\hat{y})$.
- $y_{k}$ is the true label for the *k*-th class, typically a binary value (0 or 1) indicating the correct class.
- ${\hat{y}}_{k}$ is the anticipated likelihood of the *k*-th class, as output by the model.
- $\sum_{k}$ denotes the summation over all classes *k*.
- $\log({\hat{y}}_{k})$ is the natural logarithm of the predicted probability for the *k*-th class.

The discrepancy between the expected probability distribution and the actual label distribution is measured by the cross-entropy loss. It is particularly effective in classification problems where it penalizes incorrect classifications heavily, thereby encouraging the model to improve its predictions to match the true distribution more closely.

Methods such as data augmentation, which enhances the training set by rotating and flipping the data [51], dropout, which prevents overfitting by randomly removing neurons during training [52], and batch normalization, which stabilizes and accelerates training by normalizing layer inputs [48], can significantly improve CNN performance.

## 6. Discussion and Results

### 6.1. Reconstruction of Reflectivity-Added Images

The proposed model reconstructed 3605 weapon images. This approach allows the radar system to simulate different material properties and geometrical configurations, enhancing the generalization and adaptability of the radar model by considering reflectivity-added images.

**Figure 9:**
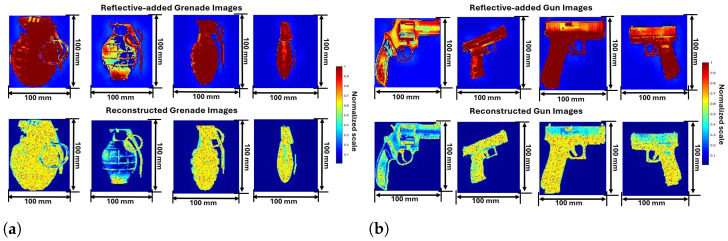
Image obtained from proposed model and reconstructed gun images from SAR (**a**) Grenades; (**b**) Guns.

**Figure 10:**
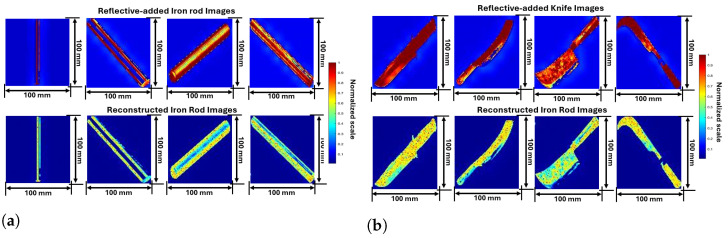
Image obtained from proposed model and reconstructed gun images from SAR (**a**) Iron Rods; (**b**) Knives.

**Figure 11:**
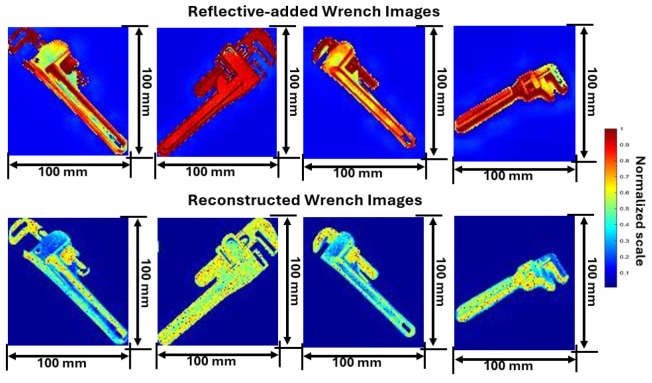
Image obtained from proposed model and reconstructed wrench images from SAR.

Thus, integrating reflectivity-added images into the SAR imaging process provides a practical solution for testing and validating the radar model’s effectiveness in real-world conditions. The analysis focuses on the comparison of reflectivity-added images and reconstructed images for various weapon images as shown in Figure 9, Figure 10 and Figure 11. This comparison highlights the efficacy of SAR imaging in capturing reflectivity properties and structural details. As discussed in Section 3, the online resource images are converted as reflectivity-added images. These reflectivity-added images becomes a target to the mm-Wave FMCW radar to reconstruct using SAR imaging technique as discussed in Section 4.

The reflectivity-added images of grenades detailed in Figure 9a highlight the metallic components with high reflectivity, revealing strong radar reflections. The reconstructed images successfully retain these high-reflectivity regions, preserving the overall shape and structural details of the grenades. The first grenade shows a clear reconstruction of its ribbed surface, accurately reflecting the intricate design. The second grenade’s cylindrical shape and safety pin are distinctly captured to maintain complex structural features. The third grenade’s oval shape and the fourth grenade’s streamlined design are both effectively reconstructed.

Images of Gun weapons with reflectivity-added are demonstrated in Figure 9b highlighting the regions with strong reflectivity in the barrel, grip, and trigger. The reconstructed images maintain these details, ensuring the guns’ overall shape and critical features are preserved. The first gun, a semi-automatic pistol, shows a precise reconstruction of its metallic barrel and polymer grip, capturing the material contrast. The second gun, another semi-automatic model, retains the intricate design of its slide and trigger. The revolver’s reconstruction is particularly notable, with the high reflectivity of the barrel and grip visible, demonstrating the technique’s effectiveness in handling different gun types. The subcompact pistol’s small size and design elements are well-preserved, highlighting the method’s capability to reconstruct fine details.

The reflectivity-added iron images are shown in Figure 10a. The first iron rod, a plain cylindrical rod, shows a uniform high reflectivity in the reconstruction, maintaining its simple design. The second rod, with a smooth metallic surface, retains its high reflectivity and smooth texture in the reconstructed image. The third rod, characterized by a threaded surface, showcases the SAR technique’s capability to capture complex surface patterns. The fourth rod, another smooth cylindrical type, demonstrates the reconstruction process’s effectiveness in preserving detailed structural elements.

Reflectivity-added images of knives highlight high reflectivity along the blade edges and metallic components as reflected in Figure 10b. The reconstructed images maintain these high reflectivity areas, accurately capturing the knives’ intricate design elements and structural details. The first knife, a combat model with a serrated edge, shows a detailed reconstruction of its blade and handle. The second knife, with a curved blade and wooden handle, retains its distinctive shape and material contrast in the reflectivity-added image. The cleaver, characterized by its wide blade, is effectively reconstructed, showcasing the model’s ability to handle diverse knife designs. The machete’s long blade and the hooked blade of the pruning knife both demonstrate the method’s robustness in capturing various blade shapes and details.

Reflectivity-added images of wrenches display high reflectivity along metallic parts such as jaws and handles. In reconstructed images as exhibited in Figure 11, these high-reflectivity areas are accurately maintained. The first wrench, an adjustable model with a red handle, shows a detailed reconstruction of its jaw and handle. The second wrench, featuring an adjustable jaw and black handle, retains its structural integrity, with the SAR technique capturing its distinctive design. The third wrench, with a wooden handle and metallic jaw, showcases the method’s ability to differentiate between materials. The fourth wrench, a fully metallic tool shows how well different design elements and material qualities can be preserved.

### 6.2. CNN Performance Results for SAR-Reconstructed Images

The methodology of machine learning technique to classify reconstructed images detailed in Section 5. The analysis of CNN algorithm for classifying the reconstructed images are comprehensively detailed in this section. The CNN model is trained on the SAR-reconstructed image dataset, which is split into 80% training and 20% testing sets to evaluate performance. The model is given 200 epochs to learn object recognition and classification using the Adam optimizer and the sparse categorical cross-entropy loss function. With a high degree of reliability in the classification results, these metrics show that the model can effectively identify true positives while minimizing false positives and negatives. The confusion matrix of the model, which displays good classification performance with low misclassification across all weapon types, further supports its efficacy. This shows the CNN is a good fit for the challenging task of analyzing SAR-reconstructed images for security applications because of its multi-layered architecture and sophisticated feature extraction capabilities.

#### Training and Evaluation

Both training and validation accuracy quickly increased during the first 10 epochs, as shown by the accuracy plot. Starting from an initial value of approximately 60%, the training accuracy quickly rises to around 90%. The validation accuracy exhibits a similar trend, indicating that the model effectively learns from the training data while also generalizing well to the validation set during these early epochs. This phase is characterized by steep learning curves, suggesting efficient optimization of the model parameters.

Beyond the 10th epoch, both training and validation accuracies converge to approximately 98% to 99%. This convergence indicates that the model has reached a high-performance level with minimal variance between the training and validation sets, signifying robust generalization. The consistent high accuracy over the remaining epochs (10–200) suggests that the model has achieved an optimal balance between learning and generalization, effectively capturing the underlying patterns in the data without overfitting.

**Figure 12:**
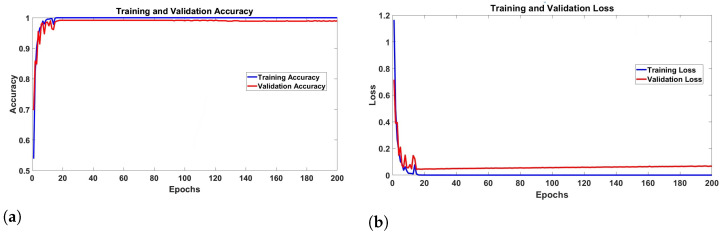
(**a**) Training and Validation Accuracy Graph and (**b**) Training and Validation Loss Graph with captions.

The training and validation accuracy across epochs illustrates the progression as shown in Figure 12a. The training accuracy curve exhibits a steady increase, indicating that the model is effectively learning from the training data. Notably, the validation accuracy curve also shows an upward trend, although with some fluctuations, suggesting that the model generalizes reasonably well to the validation set. The gap between the training and validation accuracy curves is relatively small, which is a positive indicator of the model’s ability to generalize without overfitting. This convergence implies that the chosen architecture and training regimen, including the optimizer and learning rate, are suitable for the given image classification task.

The loss plot further corroborates the observations from the accuracy plot. Initially, the training loss starts at around 1.2, rapidly decreasing to approximately 0.1 within the first 10 epochs. This significant reduction in loss reflects the model’s ability to minimize prediction errors effectively during the early stages of training. The validation loss follows a similar downward trajectory, reinforcing the conclusion that the model is learning effectively and not overfitting during the initial training phase.

After the initial rapid decline, both training and validation losses converge to near-zero values, with the training loss approaching zero and the validation loss stabilizing at a slightly higher but still minimal level. This convergence indicates that the model’s predictions are highly accurate, and the error rates are minimal. The small and stable gap between the training and validation losses suggests a well-regularized model that maintains strong generalization capabilities throughout the training process.

The training and validation loss curves, depicted in Figure 12b, demonstrate a consistent decline throughout the epochs. The training loss curve decreases smoothly, reflecting the model’s increasing proficiency in minimizing the error on the training dataset. The validation loss follows a similar downward trajectory, although it exhibits minor fluctuations, particularly in the later stages of training. This behavior is expected and indicates that the model is effectively learning the underlying patterns in the data without significant overfitting. A slight gap between training and validation loss is observed, which is typical and acceptable as long as the gap does not widen substantially, signaling overfitting.

The evaluation of training and validation metrics highlights the model’s robust learning capabilities. The consistent increase in accuracy and decrease in loss over epochs affirm the effectiveness of the CNN architecture in handling the complexity of the dataset. The small gap between training and validation metrics underscores the model’s ability to generalize, thereby ensuring its reliability for making predictions on unseen data.

**Figure 13:**
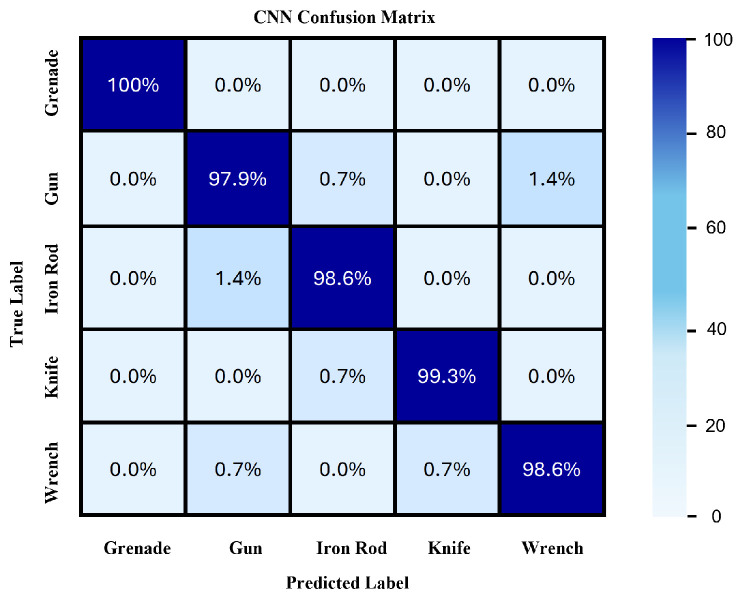
CNN Confusion Matrix Table.

The confusion matrix shown in Figure 13 demonstrates the performance of the CNN model in classifying SAR-reconstructed images of various objects. Each cell in the matrix represents the percentage of instances of an actual class (true label) that were correctly or incorrectly predicted by the model (predicted label). The diagonal cells from the top left to the bottom right indicate the correct classifications made by the model. For instance, 100% of the grenade images were correctly classified as grenades, showcasing the model’s perfect accuracy for this class. Similarly, the model correctly identified 97.9% of gun images, with a minor 1.4% misclassification where gun images were predicted as wrenches.

For iron rods, the model achieved a 98.6% accuracy, with only 1.4% misclassified as knives and guns. Knives were also classified correctly 99.3% of the time, with a 0.7% misclassification as iron rods. Wrenches saw a 98.6% correct classification rate, with 0.7% misclassified as knives and 0.7% as guns. These misclassifications, though minimal, suggest areas where the model can be further refined, potentially by improving feature extraction techniques or incorporating additional training data to enhance the model’s ability to distinguish between similar objects.

Overall, the CNN model demonstrates high accuracy across all object types, with the lowest correct classification rate being 98% for guns. The confusion matrix highlights the model’s robustness and reliability in accurately distinguishing between different objects, which is crucial for practical security and surveillance applications. The observed minor misclassifications indicate potential areas for further refinement, such as enhancing feature extraction or incorporating more diverse training data to reduce these errors. The model’s high accuracy and low misclassification rates validate the effectiveness of the SAR imaging and CNN-based classification approach in identifying various objects with high precision.

**Figure 14:**
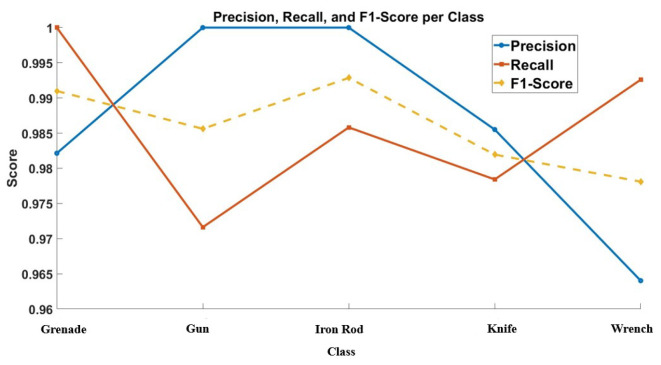
Precision, Recall and F-1 Score.

The graph in Figure 14 illustrates the precision, recall, and F1-score for each class—grenade, gun, iron rod, knife, and wrench—based on precision, recall, and F1-score metrics. The model demonstrates high precision (ranging from 0.96 to 1.00), indicating strong accuracy with few false positives across all classes. Recall values are also high (between 0.96 and 0.985), reflecting the model’s ability to correctly identify most actual instances of each weapon type. Consequently, the F1-scores, which balance precision and recall, remain robust across all categories, typically exceeding 0.97. This consistency in high scores suggests the model’s overall reliability and effectiveness in accurately distinguishing between various weapons using mm-Wave SAR images, although slight improvements could further enhance its ability to handle more challenging classifications, particularly for guns and iron rods.

In this graph, precision is consistently high across all classes, with the highest precision observed for the iron rod class. This suggests that when the model predicts an object as an iron rod, it is highly likely to be correct. Precision for grenades and guns is also robust, indicating reliable positive predictions for these classes. However, a slight dip in precision is observed for wrenches and knives, which may indicate some degree of false positive predictions.

Recall measures the ability of the model to identify all relevant instances of a class. A high recall means that most actual positives are correctly identified. The recall metric is highest for the knife class, demonstrating the model’s effectiveness in identifying knives from the dataset. Recall for grenades and iron rods is also notably high, indicating good identification rates for these objects. However, the recall for guns shows a slight decline, suggesting that some gun instances may be missed by the model. The wrench class also exhibits high recall, demonstrating effective identification.

The F1-score is the harmonic mean of precision and recall, providing a single metric that balances both concerns. A high F1-score indicates that the model has a good balance between precision and recall. The F1-scores across all classes are relatively high, with the iron rod and knife classes achieving the highest F1-scores, reflecting a strong balance between precision and recall for these objects. The grenade class also shows a high F1-score, affirming the model’s overall performance. The F1-scores for guns and wrenches are slightly lower, indicating room for improvement in balancing precision and recall for these classes.

The graph demonstrates that the CNN model performs consistently well across different object classes, achieving high precision, recall, and F1-scores. The highest precision and F1-scores for the iron rod and knife classes highlight the model’s strong performance in accurately identifying these objects. The slightly lower precision and recall for the gun class suggest that the model may benefit from additional training data or further refinement in feature extraction to improve its accuracy. Overall, the high scores across all metrics validate the effectiveness of the SAR imaging and CNN-based classification approach in identifying various objects with high precision and recall.

The CNN model exhibits robust classification performance for SAR-reconstructed images of different objects, with minor areas for enhancement. The high precision, recall, and F1-scores underscore the model’s reliability and accuracy, making it a valuable tool for applications requiring precise object identification and classification.

## 7. Comparison with Previous Works

The comparison Table 4 provides an overview of various research works that employ different radar technologies and machine-learning techniques for image reconstruction and object classification, specifically focusing on mm-Wave FMCW and SAR radars. Each study is evaluated based on parameters such as the type of radar, SAR technique, operational distance, bandwidth, data collection methodologies, reflectivity, machine learning (ML) techniques used, system complexity, type of radar configuration, CNN network size, signal processing methods, and post-processing requirements.

Previous Works 1 utilizes mm-Wave FMCW radar technology with a Range-Doppler SAR technique, operating within a distance range of 20–30 m and a bandwidth of 76–81 GHz. The study employs advanced ML techniques, specifically ResNet and VGG-19 networks, to classify objects based on radar images [2,3,4,8,22]. Given their complexity and the need for detailed feature extraction, these models are often trained for 100 to 150 epochs. Extensive real-world data collection is one of the significant attributes of this work, which helps in creating a robust dataset for training the deep learning models. However, the system complexity is high due to the advanced DSP (Digital Signal Processing) involved and the extensive post-processing requirements to enhance image quality and classification accuracy.

Previous Works 2 also leverages mm-Wave FMCW radar but with a different SAR technique, Spotlight SAR, which focuses on a narrower beam to provide high-resolution images [7,10,12,13,14,19]. The radar system operates over a broader range (10–50 m) and bandwidth (77–81 GHz). This study emphasizes using VGG-16, a similar range of 50 to 150 epochs that is typically used to train their models. These models, known for their deep architectures, usually require several epochs to achieve optimal performance, especially when trained on large or complex datasets. Additionally, early stopping criteria might also affect the final number of epochs used. and ResNet architectures for image classification, combining limited real-world data with synthetic data to enhance the training process. The system complexity is moderate, with basic DSP techniques employed, reducing the post-processing demands compared to Work 1. The use of single-material reflectivity makes this study less complex but also potentially less accurate in diverse scenarios involving multiple materials.

Previous Works 3 explores SAR Radar with a synthetic SAR technique and operates at shorter distances (5–20 m) and a slightly different bandwidth (75–80 GHz) [11,20,26,53,54,55,56]. This work primarily uses simulated data to train the machine learning models, with a focus on Support Vector Machine (SVM) and Convolutional Neural Network (CNN) techniques for classification [55,56] The system is designed to be less complex, with a lower number of CNN layers, basic DSP, and minimal post-processing requirements. The simplicity of this approach makes it more feasible for real-time applications; however, it may compromise on the depth of feature extraction and robustness of classification, particularly in more complex and cluttered environments.

This research Work stands out by integrating mm-Wave FMCW radar with a Range-Doppler SAR technique, focusing on a narrower frequency range of 77–78 GHz. This study combines both simulated and online data, which allows for a more diverse and comprehensive training dataset. By using a simpler CNN architecture with 10 layers, the proposed method balances system complexity and performance, making it suitable for real-time applications. Advanced DSP techniques are employed to ensure high-quality signal processing while minimizing post-processing requirements. The reflectivity parameters are specifically set to enhance object detection accuracy, with background reflectivity values ranging from 0 to 0.3 and object reflectivity from 0.8 to 1.

This work effectively addresses the limitations identified in previous studies by optimizing the trade-offs between system complexity, data collection, and processing requirements. The combination of a focused frequency range, efficient data collection strategy, and a streamlined CNN model demonstrates superior adaptability for real-world applications, particularly in security and surveillance scenarios. By minimizing computational demands while maintaining high classification accuracy, our approach provides a more practical and scalable solution for other advanced radar-based imaging applications, such as foliage penetration, drone-based mapping, medical imaging, etc.

## 8. Conclusions

This study demonstrates the effectiveness of integrating mm-Wave FMCW radar with Convolutional Neural Networks (CNNs) for high-resolution image reconstruction and weapon classification in security applications. By focusing on the 77–78 GHz frequency range and utilizing a Range-Doppler SAR imaging technique, our approach achieved over 98% classification accuracy, highlighting the robustness of the model in distinguishing between various weapon types. The innovative use of simulated and online data for training, along with optimized preprocessing techniques, has significantly enhanced the fidelity of reconstructed images and the overall system performance. Compared to previous works, our method offers a balanced trade-off between system complexity and computational efficiency, making it suitable for real-time. Future work will explore further optimization of the radar parameters and CNN architecture to enhance detection capabilities, as well as the expansion of the dataset to include a wider variety of objects and scenarios to validate the model’s effectiveness in diverse operational environments.

## Figures and Tables

**Table 1:** Work flow.

Step-by-Step Overview Workflow
1. Online Images Collection and Preprocessing
2. mm-Wave FMCW radar modeling and Image Reconstruction
3. Generate Reflectivity added Images
4. Reconstruct Images using SAR imaging Technique
5. Save the reconstructed images in folder
6. Import reconstructed images for classification.
7. Normalize, reshape, and design CNN Architecture
8. Performance Evaluation and Analysis

**Table 2:** Parameters of reflectivity-added images.

S.No	Parameter	Value	Property
1	Object Reflectivity	0.8 to 1	Perfect material
2	Background Reflectivity	0 to 0.3	Perfect absorber
3	Image Size	m × n	Online resource image
4	grid_size	100 × 100	Reflectivity-added image

**Table 3:** mm-Wave Parameters Considered for Modeling.

S.No	Parameter	Description	Value
1	c	Speed of light	$3\times 10^{8}$ m/s
2	max_range	Maximum range for the radar system	100 mm
3	object_range	Range of the object	50 mm
4	range_resolution	Range resolution of the radar system	3.8 mm
5	fc	Carrier frequency	77 GHz
6	B	Chirp bandwidth	$\frac{c}{2\times \mathrm{range}\_\mathrm{resolution}}$
7	Tchirp	Chirp time	$5.5\times \frac{2\times \max\_\mathrm{range}}{c}$
8	slope	Chirp slope	$\frac{B}{Tchirp}$
9	Nd	Number of scanning points in x-axis	100
10	Nr	Number of scanning points in y-axis	100
11	grid_size	Total grid size	Nd×Nr
12	step_sizex	Step size along X-axis	1.9 (λ/2)
13	step_sizey	Step size along Y-axis	1.9 (λ/2)
14	num_freq	Number of frequencies to sum over	10

**Table 4:** Comparison of This Research Work with Previous Similar Works.

Parameter	Previous Works 1 [2,3,4,8,22]	Previous Works 2 [7,10,12,13,14,19]	Previous Works 3 [11,20,26,53,54,55,56]	This Research Work
Type of Radar	mmWave FMCW	mmWave FMCW	SAR Radar	mmWave FMCW
SAR Technique	Range-Doppler SAR	Spotlight SAR	Synthetic SAR	Range-Doppler SAR
Distance	20–30 m	10–50 m	5–20 m	10–50 m
Bandwidth	76–81 GHz	77–81 GHz	75–80 GHz	77–78 GHz
Data Collection	Extensive real-world data	Limited real-world data	Simulated data	Simulated + Online data
Reflectivity	Complex multi-material	Single material	Single material	Background: 0–0.3, Object: 0.8–1
ML Technique	ResNet, VGG-19	VGG-16, ResNet	SVM, CNN	CNN
System Complexity	High	Moderate	Low	Moderate
Type of Radar	Monostatic	Bistatic	Monostatic	Monostatic
CNN Network Size	50+ layers	16–50 layers	N/A	10 layers
Signal Processing	Advanced DSP	Basic DSP	Basic DSP	Advanced DSP
Post-Processing Requirements	Extensive	Moderate	Minimal	Minimal

## Data Availability

Data are available from the authors at https://github.com/WSILABSRMAP. This can be used with due acknowledgement from the author.
